# Albuminuria after induction treatment and kidney prognosis in ANCA-associated glomerulonephritis

**DOI:** 10.1093/ckj/sfae379

**Published:** 2024-11-23

**Authors:** Aglaia Chalkia, Rachel Jones, Rona Smith, Lisa Willcocks, David Jayne

**Affiliations:** Department of Medicine, University of Cambridge, Cambridge, UK; Department of Medicine, University of Cambridge, Cambridge, UK; Vasculitis & Lupus Clinic, Addenbrooke's Hospital, Cambridge, UK; Department of Medicine, University of Cambridge, Cambridge, UK; Vasculitis & Lupus Clinic, Addenbrooke's Hospital, Cambridge, UK; Vasculitis & Lupus Clinic, Addenbrooke's Hospital, Cambridge, UK; Department of Medicine, University of Cambridge, Cambridge, UK; Vasculitis & Lupus Clinic, Addenbrooke's Hospital, Cambridge, UK

**Keywords:** albuminuria, ANCA-associated glomerulonephritis, Berden classification, end-stage kidney disease

## Abstract

**Introduction:**

It remains unclear whether persisting proteinuria in ANCA-associated glomerulonephritis (AAGN) reflects damage from the initial injury or ongoing inflammation.

**Methods:**

A retrospective, single-centre study of biopsy-proven AAGN was performed. The study defined the ‘albuminuria’ group as urine albumin-to-creatinine ratio (ACR) >300 mg/g and the ‘no albuminuria’ group as ACR ≤300 mg/g at 6 months. We sought the clinical and histopathological characteristics of both the initial and subsequent biopsies and long-term kidney outcomes stratified by albuminuria levels.

**Results:**

Two hundred and eighteen patients were included. Within the first 6 months, 28 (13%) died or progressed to end-stage kidney disease (ESKD). Among the remaining 190 patients, 37% had an ACR >300 mg/g at 6 months. The albuminuria group more frequently presented with a Berden mixed or crescentic class and had higher glomerular activity on the initial biopsy. They were more often male (OR 2.75; 95% CI 1.15–6.54), younger age (OR 0.96; 95% CI 0.93–0.99), and had fewer normal glomeruli in the biopsy (OR 0.96; 95% CI 0.93–0.99) compared with the group without albuminuria. Over the initial 5-year period, the recovery in eGFR was lower in the albuminuria group (adjusted mean difference in ΔeGFR −12.5 mL/min per 1.73 m^2^; 95% CI −15.8 to −9.1). In multivariable analysis, ACR >300 mg/g was associated with a higher risk of ESKD, even after adjusting for Berden classification and eGFR at diagnosis (hazard ratio 6.53; 95% CI 1.49–28.50).

**Conclusions:**

In a well-defined cohort of AAGN, one-third of the patients, primarily younger males with a lower percentage of normal glomeruli, had persisting albuminuria after induction treatment which was associated with worse kidney outcomes independent of Berden class and eGFR at diagnosis.

## Graphical Abstract

**Graphical Abstract:**
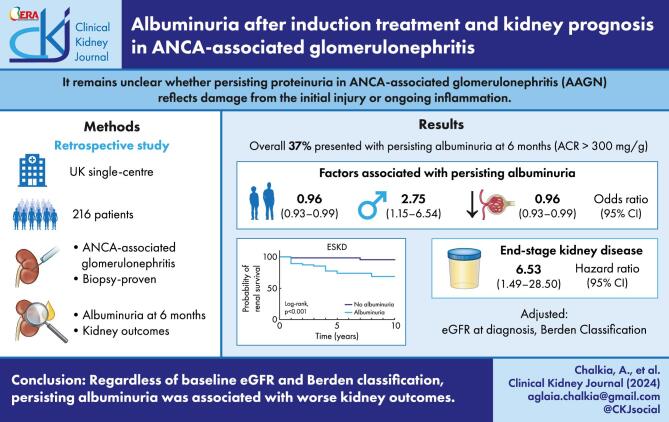

KEY LEARNING POINTS **What was known:**

- In ANCA-associated glomerulonephritis (AAGN), some patients exhibit persisting proteinuria despite induction treatment aimed at achieving remission.
- Proteinuria has been associated with worse kidney outcomes.
- It is unclear whether persistent proteinuria reflects damage from the initial injury or ongoing inflammation in AAGN.

**This study adds:**

- Younger males and patients with fewer normal glomeruli in the initial biopsy were more likely to exhibit persisting albuminuria beyond 6 months.
- Persisting albuminuria correlated with glomerular inflammation rather than fibrosis at baseline biopsy.
- Regardless of baseline eGFR or Berden classification, persisting albuminuria was associated with worse kidney outcomes.

**Potential impact:**

- This study highlights the importance of monitoring albuminuria beyond 6 months in managing AAGN, serving as a predictor of adverse kidney outcomes.
- This underscores the necessity for targeted and intensive interventions aimed at improving long-term kidney outcomes.

## INTRODUCTION

ANCA (antineutrophil cytoplasmic antibody)-associated vasculitides (AAV) are characterized by small-vessel necrotizing inflammation and are categorized into the clinical phenotypes microscopic polyangiitis (MPA), granulomatosis with polyangiitis (GPA), and eosinophilic granulomatosis with polyangiitis (EGPA). AAV can present with multi-organ involvement and kidneys are commonly affected, especially in MPA (90%–100%) and GPA (50%–80%) [1]. Despite advances in the treatment and diagnosis of AAV [2], many patients present with severe kidney dysfunction and 20%–40% progress to end-stage kidney disease (ESKD) in the first 5 years [3, 4]. A predominant histological pattern on kidney biopsy is segmental necrotizing glomerulonephritis, affecting the glomerular capillaries. Following the disruption of the glomerular capillary, circulating cells, inflammatory mediators, and plasma proteins pass through the capillary wall into the Bowman space, leading to the development of crescents. In addition to these active lesions, glomeruli may also exhibit global or segmental sclerosis lesions [5]. Beyond the glomerular changes, ANCA-associated glomerulonephritis (AAGN) can also involve the interstitial and tubular area characterized by inflammation and/or fibrosis, being more dominant in cases that are associated with myeloperoxidase (MPO)-ANCA [6].

Kidney involvement in AAV often presents as a rapid decline in kidney function, known as rapidly progressive glomerulonephritis [7]. Patients may exhibit varying degrees of proteinuria and haematuria. In kidney diseases, the level of proteinuria is closely linked to a damaged glomerular filtration barrier, which can display either inflammatory or fibrotic features [8]. This barrier consists of the glomerular endothelium, glomerular basement membrane (GBM), and the highly specialized podocytes on the urinary side of the GBM. The degree of proteinuria reflects the extent of glomerular lesions, and its persistence is associated with a more rapid decline in estimated glomerular filtration rate (eGFR). Studies on AAGN have mainly focused on kidney dysfunction [9, 10]. Following induction treatment aimed at achieving remission, as assessed by the Birmingham Vasculitis Activity Score (BVAS), some patients still manifest persisting proteinuria [11]. However, it remains uncertain whether this persistent proteinuria reflects damage of the initial injury or an ongoing inflammation that has not been effectively treated. In this study, focused on a well-defined cohort of patients with biopsy-proven AAGN, we aimed to investigate the association between persistent albuminuria beyond 6 months and the histopathological injury in both the initial and subsequent biopsies. We also sought to assess whether this albuminuria might have an impact on long-term kidney function.

## MATERIALS AND METHODS

### Patients

**Figure 1:**
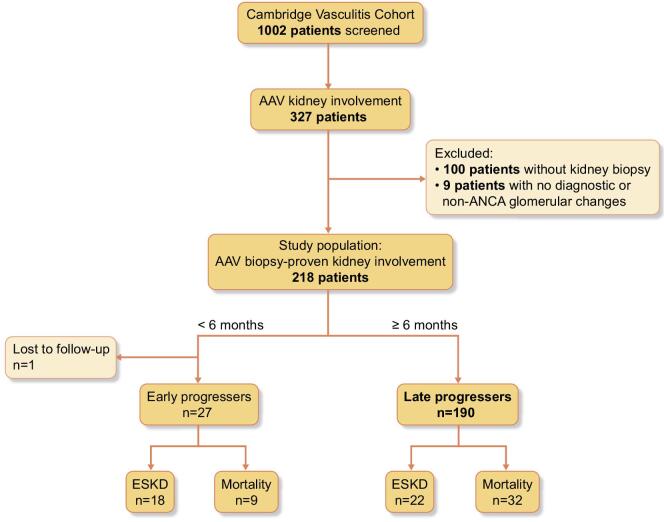
Flow chart of the whole cohort. ESKD, end-stage kidney disease; AAV, ANCA-associated vasculitis.

This study used data obtained from the AAV cohort at Cambridge University Hospitals NHS Foundation Trust. We retrospectively investigated 1008 patients diagnosed between November 2002 and December 2022. Among the 327 patients with kidney involvement, we included all the patients with biopsy-proven AAGN, excluding patients with no diagnostic kidney biopsy or those with non-AAV glomerular pathologies, such as overlap glomerular diseases or diabetic nephropathy (Fig. 1).

In accordance with the UK National Health Service Research Ethics Committee guidelines, ethics approval was not required as this work comprised anonymous retrospective data and all treatment decisions were made prior to our evaluation.

### Data collection

We collected age at diagnosis, gender, disease diagnosis, ANCA serotype, details of induction and maintenance treatment, and eGFR (mL/min per 1.73 m^2^ according to the CKD-EPI 2021 formula). Urine assessment was performed by measurement of albumin-to-creatinine ratio (ACR) (mg/g) by a morning spot urine sample or dipstick urine and haematuria (>1+ by urine dipstick, or ten or more red blood cells per high-power field) confirmed by two measurements. The ‘albuminuria’ group was defined as ACR >300 mg/g at 6 months and the ‘no albuminuria’ group was defined as ACR ≤300 mg/g or two consecutive dipstick urine measurements showing negative or trace for albumin at 6 months [12].

The increase in eGFR (ΔeGFR) over time was calculated, which was defined as the difference between the eGFR at that time point and eGFR at baseline. Kidney survival time for each patient was computed from baseline evaluation at the time of biopsy to the last time of follow-up or the time point of reaching ESKD. ESKD was defined as eGFR <15 mL/min per 1.73 m^2^ or on haemodialysis for >3 months or kidney transplantation.

### Kidney biopsies

Kidney biopsies were performed according to clinical practice and assessed by a histopathologist experienced in nephropathology. The histopathological classification used the Berden classification [5] as follows: focal, ≥50% normal glomeruli; crescentic, ≥50% glomeruli with cellular crescents; mixed, <50% normal, <50% crescentic, <50% globally sclerotic glomeruli; and sclerotic, ≥50% globally sclerotic glomeruli. We also recorded the severity grade of interstitial fibrosis and tubular atrophy, which were quantified in the renal cortex, based on Banff terms [absent (≤5%), mild (6%–25%), moderate (26%–50%), severe (>50%)] and the presence of tubulitis, acute tubular injury, arteritis, and arteriosclerosis (degree of intimal thickening). Kidney biopsies with fewer than eight glomeruli or medulla only were considered inadequate.

### Statistical analysis

Continuous variables were presented as mean (± standard deviation) for normally distributed data and as median (interquartile range, IQR) for non-parametric distributions and were compared with the *t*-test or the Mann–Whitney test, where appropriate. Categorical variables, presented as *n* (%), were compared with the *χ*^2^ test. Kidney survival analysis was performed with the Kaplan–Meier method censored by death or loss to follow-up and differences between categories were determined using the log-rank test. Univariate logistic regression analysis was used to assess the impact of various clinical elements on the probability of persistent albuminuria beyond 6 months. The changes between first biopsy and repeat biopsy were tested by the Wilcoxon signed-rank test. Odds ratios (ORs) were reported with their respective 95% confidence intervals (CIs) and statistical significance. All independent variables that showed statistical significance on univariate analysis were entered in a multivariate logistic regression model. ΔeGFR was analysed using mixed effects models for repeated measures with albuminuria group, time, and albuminuria-by-time interaction as factors, and baseline eGFR and age as covariates. A Cox regression model was used to compare the hazard ratios (HRs) for the renal endpoint, ESKD, between the albuminuria groups. A sensitivity analysis was conducted for the combined endpoint of ESKD or death. The complete case method was adopted, with no imputation of missing data. The threshold of statistical significance was set as *P* < 0.05 (two-tailed). Data were analysed using the Statistical Package for the Social Sciences (SPSS) software program, version 25.0 for Windows (SPSS, Chicago, IL, USA) and GraphPad Prism (version 10.1.0).

## RESULTS

### Clinical characteristics at baseline in the total cohort

Two hundred and eighteen patients with biopsy-proven AAGN were included. Within the first 6 months, 27 patients either died or progressed to ESKD; they were categorized as ‘early progressers’ (Fig. 1). The remaining 190 patients, of whom 42% were female, were categorized as ‘late progressers’. This group had a median age of 67 years (IQR 56–74.2). Fifty-five per cent were MPO-ANCA positive, 43% proteinase 3 (PR3)-ANCA-positive, and 2% negative (confirmed by kidney biopsy). All the cases were *de novo* AAGN. Notably, kidney involvement was the initial manifestation of AAV in all but five patients, who had relapsed AAV without any prior kidney involvement. At the time of diagnosis, median eGFR was 25 mL/min per 1.73 m^2^ (IQR 12–45) and median ACR was 640 mg/g (IQR 190–1730). The most prevalent histopathological class was focal, observed in 47% of the cases. Regarding the induction treatment, 56% received cyclophosphamide, 19% rituximab, and 25% combination treatment with rituximab and cyclophosphamide. For maintenance treatment, 46% received rituximab, 39% azathioprine, and 13% mycophenolate mofetil.

### Persistent albuminuria beyond 6 months

#### Clinical parameters

Following the first 6 months of induction treatment, 64 patients (37%) had albuminuria (defined as ACR >300 mg/g). They demonstrated more severe kidney dysfunction at the time of diagnosis compared with patients without albuminuria at 6 months (median eGFR 16 versus 31 mL/min per 1.73 m^2^, *P* = .001, and ACR 1750 versus 270 mg/g, *P* < .001). Furthermore, the albuminuria group experienced a higher rate of progression to ESKD (23% versus 2%, *P* < .001). Younger and male patients more frequently had albuminuria at 6 months (both *P* < .05). In contrast, no differences were observed in the co-occurrence of haematuria beyond 6 months between the groups, and the persistence of albuminuria was not influenced by ANCA serotype (Table 1, Supplementary Table S1).

**Table 1:** Clinical characteristics of the late progressers cohort with comparison between groups with or without albuminuria at 6 months.

		Albuminuria^b^		
Characteristics	Late progressers^a^ *N* = 190	Yes *N* = 64	No *N* = 111	*P* value
Demographics				
Female, % (*n*)	42 (79)	32 (20)	47 (50)	**0.038**
Age (years), median (IQR)	67 (56–74)	63 (52–76)	70 (62–74)	**0.030**
Serological phenotype				0.843
MPO, % (*n*)	55 (105)	53 (34)	56 (63)	
PR3, % (*n*)	43 (82)	45 (29)	42 (47)	
Negative, % (*n*)	2 (3)	2 (1)	1 (1)	
Presentation				0.652
Newly diagnosed AAV, % (*n*)	97 (185)	98 (63)	96 (107)	
Relapsed, AAV % (*n*)	3 (5)	2 (1)	4 (4)	
Clinical phenotype				0.718
MPA, % (*n*)	57 (109)	61 (39)	59 (65)	
GPA, % (*n*)	40 (76)	36 (24)	38 (42)	
EGPA, % (*n*)	3 (5)	2 (1)	4 (4)	
Organ involvement				
Lung, % (*n*)	52 (91)	43 (26)	53 (53)	0.418
ENT, % (*n*)	32 (61)	33 (20)	33 (35)	1
Eyes, % (*n*)	9 (17)	7 (4)	11 (12)	0.418
Skin, % (*n*)	8 (16)	8 (5)	10 (11)	0.788
Heart, % (*n*)	2 (3)	2 (1)	2 (2)	1
Gastrointestinal % (*n*)	5 (9)	5 (3)	6 (6)	1
Peripheral Nervous System % (*n*)	9 (17)	3 (2)	15 (16)	**0.019**
Baseline parameters				
eGFR, mL/min per 1.73 m^2^, median (IQR)	25 (12–45)	16 (9–35)	31 (18–54)	**0.001**
ACR, mg/g, median (IQR)^c^	640 (190–1730)	1750 (850–2570)	270 (122–665)	**<0.001**
Haematuria, % (*n*)^d^	97 (176)	98 (61)	96 (99)	0.651
Berden classification^e^				**<0.001**
Focal, % (*n*)	47 (88)	19 (12)	64 (70)	
Mixed, % (*n*)	29 (54)	40 (25)	24 (26)	
Crescentic, % (*n*)	17 (32)	30 (19)	7 (8)	
Sclerotic, % (*n*)	7 (14)	11 (7)	5 (6)	
ANCA renal risk score^f^				**<0.001**
Low, % (*n*)	43 (81)	24 (12)	61 (63)	
Medium, % (*n*)	39 (74)	63 (32)	34 (35)	
High,% (*n*)	8 (15)	14 (7)	6 (6)	
At 6 months parameters				
eGFR, mL/min per 1.73 m^2^, median (IQR)	45 (30–64)	32 (19–52)	50 (35–69)	**0.001**
ACR, mg/g, median (IQR)^g^	130 (10–540)	790 (480–1720)	50 (0–120)	**<0.001**
ACR <30 mg/g, % (*n*)	14 (20/144)	0 (0/144)	14 (20/144)	
ACR 30–300 mg/g, % (*n*)	43 (61/144)	0 (0/144)	43 (61/144)	
ACR >300 mg/g, % (*n*)	44 (63/144)	44 (63/144)	0 (0/144)	
Haematuria % (*n*)^h^	31 (54)	35 (21)	25 (26)	0.215
Induction treatment^i^				0.122
RTX + CYC, % (*n*)	25 (45)	28 (18)	23 (26)	
CYC, % (*n*)	56 (100)	55 (35)	50 (55)	
RTX, % (*n*)	19 (35)	14 (9)	22 (24)	
Maintenance treatment^j^				0.568
RTX, % (*n*)	46 (84)	43 (26)	49 (51)	
AZA, % (*n*)	39 (72)	48 (29)	34 (36)	
MMF, % (*n*)	13 (24)	8 (5)	17 (18)	
Follow-up (years), median (IQR)	5 (2–8)	4 (2–7)	5 (3–8)	0.089
ESKD, % (*n*)	12 (22)	23 (15)	2 (2)	**<0.001**
Mortality, % (*n*)	17 (32)	19 (12)	15 (16)	0.528

RTX, rituximab; CYC, cyclophosphamide; AZA, azathioprine; MMF, mycophenolate mofetil; bold, p<0.05.

The group ‘albuminuria’ was defined as ACR >300 mg/g at 6months.

a Early progressers (28 patients) were excluded.

b Fifteen missing values for ACR after induction treatment.

c Sixteen missing values.

d Seven missing values.

e Two missing values.

f Twenty missing values.

g Fifteen missing values and 31 semi-quantitative values (by stick urine negative/trace albumin).

h Six missing values.

^i^Eight missing values in both groups.

j Ten missing values in both groups.

#### Histological parameters

**Figure 2:**
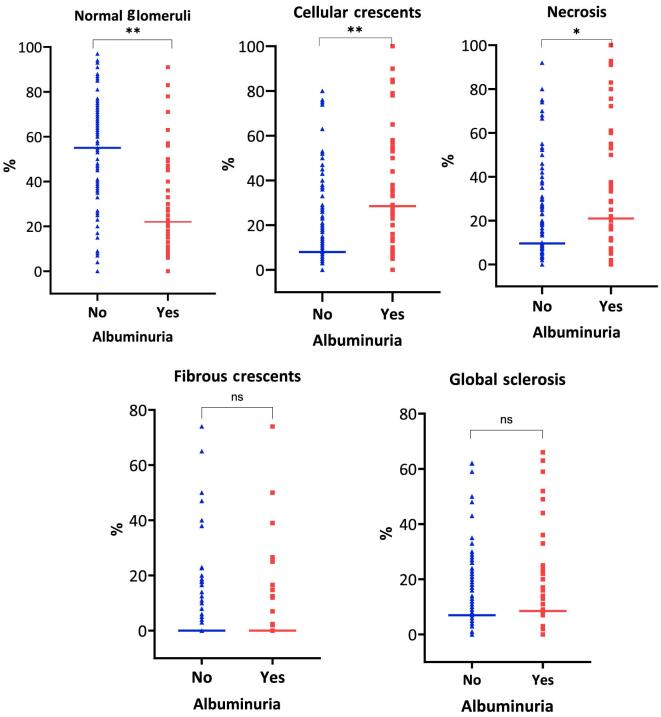
Comparison between glomerular histological features and groups with or without albuminuria at 6 months. Albuminuria, ACR at 6 months; No, no albuminuria, defined as ACR ≤300 mg/g; Yes, albuminuria defined as ACR >300 mg/g; ns, non-significant; *P < 0.05, **P < 0.001. Aligned dot plots represent medians and IQR with individual data points.

At diagnostic biopsy, patients with albuminuria at 6 months were more likely to have a Berden mixed (40% versus 24%, *P* = .029) or crescentic class (30% versus 7%, *P* < .001) compared with those without albuminuria. Additionally, patients with albuminuria had higher levels of glomerular activity, characterized by the proportion of glomeruli with cellular crescents (28% versus 8%, *P* = .001) and necrosis (22% versus 10%, *P* = .036) and a lower percentage of normal glomeruli (22% versus 57%, *P* < .001) in their initial kidney biopsies when compared with those without persistent albuminuria. It is worth noting that similar rates between the two groups were observed in fibrotic features, including global sclerosis, interstitial fibrosis/tubular atrophy, and arteriosclerosis (Fig. 2, Supplementary Fig. S1).

### Predictors of albuminuria beyond 6 months

Baseline parameters, including younger age, male gender, lower eGFR at diagnosis, higher glomerular activity (cellular crescents/necrosis), and lower normal glomeruli, predicted albuminuria at 6 months in univariate logistic regression analysis. When these parameters were included as independent variables in a multivariate model, it showed that younger age, male gender, and lower normal glomeruli held predictive value when considered together. However, lower eGFR at diagnosis was not predictive in this model. Additionally, other histopathological fibrotic features, such as, interstitial fibrosis and global sclerosis, choice of induction treatment, or ANCA type or co-occurrence of haematuria, did not demonstrate predictive value in the univariate analysis (Table 2).

**Table 2:** Baseline factors predicting albuminuria at 6 months after remission induction treatment for ANCA-associated glomerulonephritis.

	Albuminuria			
	Univariate logistic regression		Multivariate logistic regression	
Variables	OR (95% CI)	*P* value	OR (95% CI)	*P* value
Gender (male vs female)	**2 (1.05–3.83)**	**0.034**	**2.75 (1.15–6.54)**	**0.022**
Age, years	**0.97 (0.95–0.99)**	**0.02**	**0.96 (0.93–0.99)**	**0.046**
ANCA (MPO vs PR3)	0.54 (0.33–8.90)	0.666		
Baseline				
eGFR	0.98 (0.96–0.99)	**0.003**	0.99 (0.97–1.01)	0.427
Haematuria at diagnosis	3.02 (0.34–26.46)	0.318		
Berden class				
Focal (reference)	Reference		Reference	
Mixed	6.10 (2.56–14.55)	**<0.001**	1.76 (0.43–7.21)	0.429
Crescentic	15.52 (5.35–45.01)	**<0.001**	2.90 (0.36–22.97)	0.313
Sclerotic	4.6 (1.10–19.19)	**0.036**	0.87 (0.10–6.98)	0.897
Cellular crescents (%)	1.02 (1.01–1.04)	**<0.001**	0.99 (0.96–1.02)	0.802
Necrosis (%)	1.01 (1.00–1.03)	**0.006**	0.99 (0.98–1.01)	0.730
Normal glomeruli (%)	**0.95 (0.94–0.97)**	**<0.001**	**0.96 (0.93–0.99)**	**0.013**
Global sclerosis (%)	1.00 (0.98–1.02)	0.582		
Interstitial fibrosis				
Absent % (*n*) (reference)	Reference			
Mild % (*n*)	0.87 (0.37–2.07)	0.764		
Moderate % (*n*)	1.26 (0.41–3.80)	0.680		
Severe % (*n*)	1.35 (0.39–4.37)	0.655		
Induction treatment				
RTX (reference)	Reference			
CYC	1.69 (0.70–4.07)	0.236		
RTX + CYC	1.84 (0.69–4.88)	0.217		

ANCA, antineutrophil cytoplasmic antibody; RTX, rituximab; CYC, cyclophosphamide; bold, p<0.05.

The group ‘albuminuria’ was defined as ACR >300 mg/g at 6 months.

### Kidney re-biopsy data

In the albuminuria group, 11 patients underwent a repeat kidney biopsy due to suspected disease flare or for prognostic reasons, and they continued to present proteinuria at that time point. The median time between the initial biopsy and the repeat biopsy was 18 months (IQR 8–89). In the repeat biopsy, there was evidence of ongoing histological activity, cellular crescents, and/or necrosis, in a median 13% of the glomeruli, accounting for 36% of all biopsies (4/11). However, this activity appeared less pronounced than in the initial biopsy. Concurrently, there was an overall increase in fibrotic features, such as global and segmental sclerosis and interstitial fibrosis. Interestingly, the proportion of normal glomeruli remained consistent between the biopsies. Regarding the Berden classification, 40% of the repeat biopsies showed a transition from focal or crescentic to mixed or sclerotic classes (Table 3).

**Table 3:** Histopathological characteristics of paired initial and repeat biopsies in the albuminuria group.

	Albuminuria group		
Characteristics	Initial biopsy *N* = 11	Repeat biopsy *N* = 11	*P* value
Time internal (months), median (IQR)		18 (8–89)	
Berden classification ^a^			**0.34**
** **Focal class % (*n*)	10 (1)	0 (0)	
** **Mixed class % (*n*)	40 (4)	45 (5)	
** **Crescentic class % (*n*)	30 (3)	0 (0)	
** **Sclerotic class % (*n*)	20 (2)	55 (6)	
Glomeruli			
** **Normal glomeruli (%), median (IQR)	18 (10–38)	25 (15–50)	0.386
** **Global sclerosis (%), median (IQR)	3 (0–43)	36 (23–62)	**0.012**
** **Segmental sclerosis (%), median (IQR)	0 (0–12)	19 (9–34)	**0.007**
** **Cellular crescents (%), median (IQR)	38 (6–58)	0 (0–4)	**0.032**
** **Fibrinoid necrosis (%), median (IQR)	18 (0–44)	0 (0–0)	**0.038**
** **Fibrous crescents (%), median (IQR)	0 (0–20)	0 (0–0)	0.593
Interstitial/tubular area			
** **Fibrosis/atrophy			0.882
** **Absent % (*n*)	67 (6)	0 (0)	
** **Mild % (*n*)	22 (2)	22 (2)	
** **Moderate % (*n*)	11 (1)	33 (3)	
** **Severe % (*n*)	0 (0)	44 (4)	
** **Tubulitis % (*n*)	20 (2)	9 (1)	
** **Acute tubular injury % (*n*)	40 (4)	0 (0)	
**Vessels**			
** **Arteritis % (*n*)	0 (0)	0 (0)	*n*/a
** **Arteriosclerosis % (*n*)	45 (5)	45 (5)	0.524

*n*, number; n/a, not available; bold, p<0.05.

The group ‘albuminuria’’ was defined as ACR >300 mg/g at 6 months.

a One missing value for Berden classification.

### Kidney survival stratified by albuminuria and Berden classification

**Figure 3:**
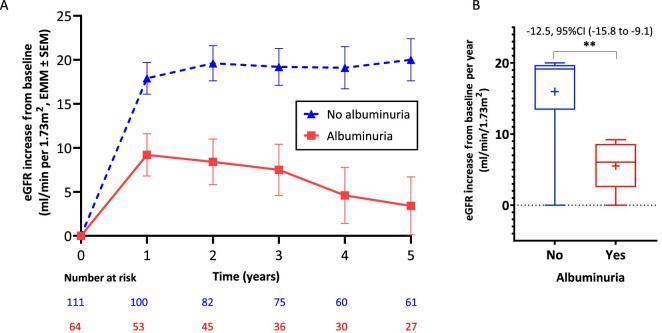
Increase in eGFR from baseline among late progressers cohort. (**A**) Estimated marginal means (EMMs) ± SEM increase in albuminuria group over 5 years. (**B**) Increase from baseline per year over 5 years obtained by mixed effects model for repeated measures analysis with the fixed effects of albuminuria group, time, albuminuria and time interaction as factors, and baseline eGFR and age as covariates. Box and whisker plot: the line represents the median value, + indicates mean, the length of the box reflects the IQR, and the T-bars represent maximum and minimum values. **P < 0.001. Albuminuria, ACR at 6 months; 0, ACR ≤300 mg/g; 1, ACR >300 mg/g.

Over the initial 5-year period, the albuminuria group presented a mean eGFR increase that was 6.6 mL/min per 1.75 m² (95% CI 17.1–21.1) lower compared with the no albuminuria group, which recorded a mean eGFR increase of 19.1 mL/min per 1.75m² (95% CI 3.9–9.3) (adjusted for age and baseline eGFR; difference −12.5 mL/min per 1.73 m²; 95% CI −15.8 to −9.1; *P* < 0.001) (Fig. 3). In the subgroup of patients with quantitative ACR measurements (Supplementary Table S2), patients with an ACR >300 mg/g showed a reduced eGFR increase compared with those with an ACR <30 or 30–300 mg/g. The latter two groups exhibited similar rates of eGFR increase (Supplementary Fig. S2).

**Figure 4:**
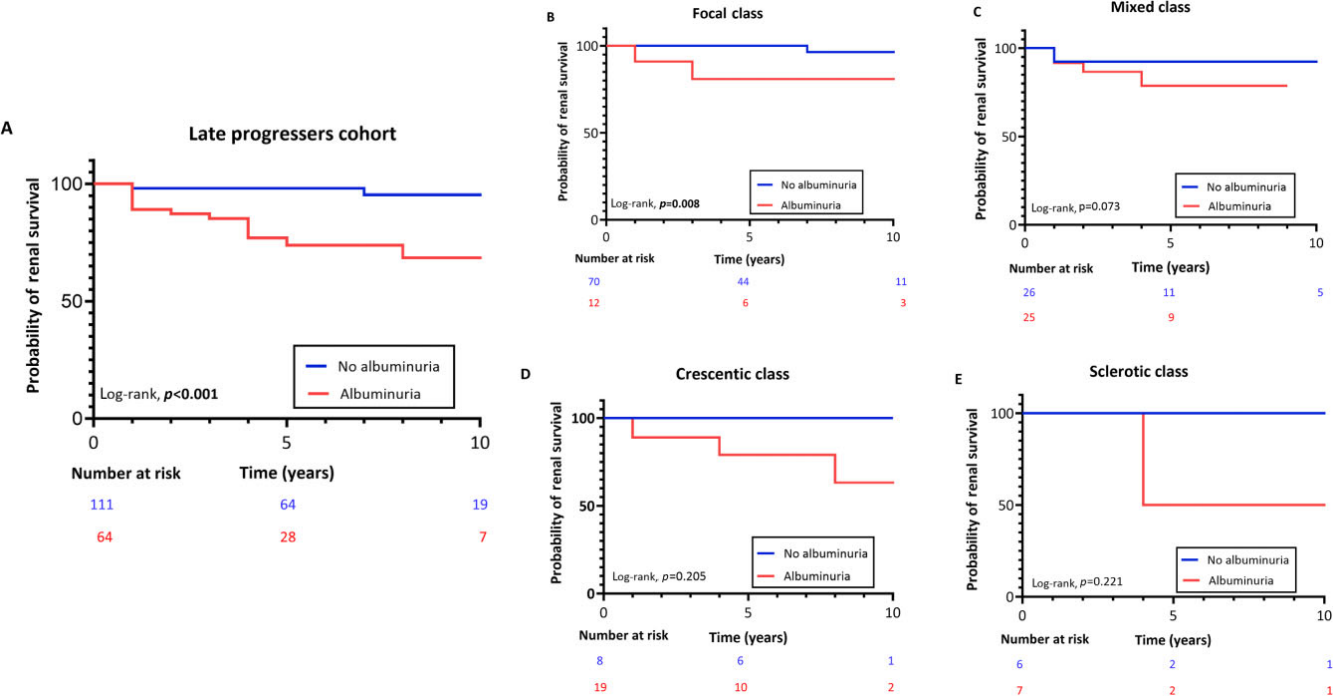
(**A**) Kaplan–Meier analysis of ESKD in the late progressers cohort according to the presence or absence of albuminuria at 6 months. (**B**–**E**). Kaplan Meier analysis of ESKD in the late progressers cohort according to Berden classification stratified by the presence or absence of albuminuria at 6 months.

Over the median follow-up of 5 years (IQR 2–8), in the late progressers cohort 22 patients developed ESKD and 32 died. Fifteen ESKD events occurred in those with albuminuria while only two occurred in those without. The cumulative ESKD-free survival rates at 3 and 5 years were lower in the group with albuminuria (log-rank, *P* = 0.001) (Fig. 4). We assessed long-term kidney survival based on the Berden classification and the presence or absence of albuminuria at 6 months. For those with albuminuria, kidney survival rates at 3 and 5 years were: focal class, 80%; mixed class, 85% at 3 years and 80% at 5 years; crescentic class, 90% at 3 years and 80% at 5 years; and sclerotic class, 100% at 3 years and 50% at 5 years. In comparison, patients without albuminuria experienced favourable 3- and 5-year kidney survival rates, exceeding 90% across all the Berden classes (Fig. 4). In the subgroup of patients with quantitative ACR measurements (Supplementary Table S2), patients with an ACR >300 mg/g had reduced renal survival compared with those with an ACR <30 or 30–300 mg/g, both of which exhibited similar curves (Supplementary Fig. S3).

### Multivariable Cox proportional hazard analyses for end-stage kidney disease

Univariable analysis was performed to identify risk factors for ESKD (Supplementary Table S3) in the late progressers cohort. Factors predictive for ESKD were lower eGFR at diagnosis, albuminuria at 6 months, type of induction treatment, and proportion of normal glomeruli and global sclerosis. Table 4 presents the multivariable-adjusted hazard ratios for predicting ESKD among different albuminuria groups. After adjusting for age, baseline eGFR, Berden classification, and the proportion of normal glomeruli, the albuminuria group was associated with poorer kidney survival [(HR 6.53; 95% CI 1.49–28.50; *P* = 0.013; model 1), (HR 6.33; 95% CI, 1.74 to 22.94; *P* = 0.005; model 2), (HR 4.79; 95% CI, 1.31–17.50; *P* = 0.018)].

**Table 4:** Multivariable Cox proportional hazard analyses for kidney survival (end-stage kidney disease) in the late progressers cohort.

	Adjusted model 1 (14 events)		Adjusted model 2 (17 events)		Adjusted model 3 (14 events)	
Variable	HR (95% CI)	*P* value	HR (95% CI)	*P* value	HR (95% CI)	*P* value
Albuminuria	**6.53** **(1.49–28.50)**	**0.013**	**6.33** **(1.74–22.94)**	**0.005**	**4.79** **(1.31–17.50)**	**0.018**
eGFR at diagnosis	**0.93** **(0.88–0.98)**	**0.019**	**0.94** **(0.90–0.99)**	**0.025**	**0.94** **(0.89–1.00)**	**0.048**
Age			1.02 (0.98–1.05)	0.255		
Berden classification						
** **Focal (reference)	Reference					
** **Mixed	**0.74** **(0.15–3.59)**	**0.712**				
** **Crescentic	**0.43** **(0.07–2.52)**	**0.355**				
** **Sclerotic	**0.65** **(0.06–6.90)**	**0.726**				
Normal glomeruli					0.98 (0.95–1.01)	0.379

Albuminuria was defined as urine ACR >300 mg/g at 6 months; bold, p<0.05.

In a sensitivity analysis, albuminuria remained associated with the combined risk of ESKD or death when adjusted for age and eGFR at diagnosis. However, it was not associated with histological parameters (Supplementary Tables S4 and S5).

## DISCUSSION

In a well-defined biopsy proven AAGN cohort, we observed a prevalence of 37% for albuminuria persisting beyond 6 months following induction treatment. Notably, younger males, hinting at potential undertreatment within this subgroup using current dosing regimens, and patients with a lower percentage of normal glomeruli, indicating more extensive disease, in the initial biopsy were more likely to experience this outcome. In this group, there was a higher occurrence of increased glomerular activity compared with fibrotic features, indicating an association with glomerular inflammation rather than glomerular fibrosis at baseline. The ΔeGFR over the first 5 years was different between the albuminuria and no albuminuria groups, with the risk of ESKD higher in the albuminuria group. These findings remained consistent regardless of the baseline kidney function or Berden classification, highlighting the clinical significance of albuminuria at 6 months as a predictor of adverse kidney outcomes.

Albuminuria is a well-established marker in glomerular diseases such as lupus nephritis and IgA nephropathy, and its trajectory is a key component in assessing treatment response criteria [12, 13]. In the ADVOCATE trial, the avacopan group [14] had earlier reduction of albuminuria (albumin-to-creatinine ratio) and better recovery of eGFR and this improvement continued until 12 months. These findings indicate that although patients are defined as having achieved a vasculitis remission, usually between 3 and 6 months, current remission definitions are inadequate and true remission of kidney disease may take longer or not occur at all. After treatment, cellular crescents either fully or partially resolve or progress to fibrous formations, ultimately leading to segmental or global glomerulosclerosis. Due to limited data on repeat biopsies in AAGN and the variability in immunosuppressive treatment, the exact timeline of these changes remains unknown. Two protocol biopsies, one after a year on cyclophosphamide and another after 4 months of induction treatment, showed reduced but persistent histopathological activity, indicating incomplete resolution [15, 16]. In line with these observations, our cohort analysis revealed that patients with albuminuria at 6 months more frequently had higher glomerular activity and lower normal glomeruli at baseline, whereas histopathological fibrotic features, such as global sclerosis, interstitial fibrosis/tubular atrophy, and arteriosclerosis, did not demonstrate any difference. Higher levels of proteinuria at diagnosis have been associated with histopathological parameters, revealing a strong association with fewer normal glomeruli [17]. Despite varying time intervals between subsequent biopsies, limited yet persistent glomerular activity, increasing fibrotic changes, and a constant percentage of normal glomeruli were observed. These observations suggest that albuminuria at 6 months was indicative of ongoing glomerular inflammation, contributing to fibrosis with sustained low activity, and are consistent with the finding of another study which revealed that the persisting proteinuria is associated with subsequent renal relapse [11].

Notably, kidney involvement in AAV can co-occur with non-AAV glomerular pathologies, making it challenging to assess the distinct impact of albuminuria in AAGN without histological confirmation. In a *post hoc* analysis of the RAVE and WGET trials, which included 147 patients with kidney involvement achieving remission (including 37 with available kidney biopsy), 43% of these patients exhibited persistent proteinuria beyond 6 months, as measured by urine dipstick [18]. However, in contrast to our cohort, this group did not experience worse kidney outcomes, such as ESKD or slope of change of eGFR. In a *post hoc* analysis including 441 patients with kidney involvement, the prevalence of persistent proteinuria, measured by urine protein-to-creatinine ratio, was 34.3% [11]. Consistent with our cohort, proteinuria >0.05 g/mmol (equivalent to protein-to-creatinine ratio 500 mg/g and albumin-to-creatinine ratio 300 mg/g) was identified as a risk factor for adverse kidney outcomes, including a higher risk of ESKD, death, and kidney relapse. In this study, the reported rates of proteinuria in 65 patients with available kidney biopsy were as follows: 22.7% in the focal class, 73.3% in the crescentic class, 52.9% in the mixed class, and 88.9% in the sclerotic class. Interestingly, in our cohort the prevalence of albuminuria in the sclerotic class was lower (54%), although we acknowledge the limited number of sclerotic class kidney biopsies in this study.

While in AAGN the persistence of haematuria has garnered attention as a potential indicator of ongoing kidney inflammation and as a predictor of renal relapse [11, 19, 20], there are inconsistent results from kidney biopsies [21]. Furthermore, activity and damage scoring tools (BVAS and VDI respectively) bias towards haematuria being considered a marker of disease flare and proteinuria being a marker for kidney damage. In our cohort, we observed a similar prevalence of persistent haematuria in both albuminuria groups, and we found no evidence suggesting a link between persistent haematuria and worse kidney progression. Despite previous reports associating MPO-ANCA with more severe kidney dysfunction and higher albuminuria, our study did not reveal a substantial impact of ANCA type on proteinuria persistence at 6 months [22].

The limitations of our study stem from its retrospective nature. Attributing albuminuria to long-term kidney outcomes is further complicated by the diversity of immunosuppressive treatments received and variations in the duration of maintenance treatment. Moreover, the low incidence of ESKD events limited our ability to assess the risk while accounting for additional confounding factors. We did not consider kidney relapse in our analysis, as not all cases were histologically confirmed. Additionally, due to the retrospective nature of the study and the long follow-up during which variable changes in the treatment were implemented, we did not collect data on antiproteinuric drugs such as RAASi and SGLT2i, as assessing their impact would be challenging. Another potential limitation lies in our histological method of assessing the presence of proteinuria at 6 months using initial biopsies rather than protocol biopsies at 6 months. However, the literature has limited data on subsequent biopsies, and our study contributes valuable information in this context. All AAGN cases were histopathologically confirmed, excluding biopsies with overlapping changes affecting proteinuria progression. Data from a single centre ensure homogeneity in albuminuria measurement and kidney histopathological evaluations. Despite these limitations, our study provides valuable insights, featuring a relatively long-term follow-up period exceeding durations in many other studies.

The primary goal in managing AAV with kidney involvement is achieving and sustaining remission, preventing irreversible damage, and avoiding both under- and overtreatment. Notably, AAV is still associated with a high ESKD rate compared with most other glomerular diseases, partially attributed to the abrupt and severe glomerular activity presentation. Nevertheless, some patients may present a grumbling kidney disease that has not been effectively treated. The persistence of albuminuria beyond 6 months serves as an indicator of ongoing kidney disease activity. This study sheds light on the importance of monitoring albuminuria beyond the 6 months in the management of AAGN, revealing its potential in identifying patients at higher risk of progressing to ESKD. This emphasizes the necessity for targeted and intensive interventions to improve long-term kidney outcomes.

## Supplementary Material

sfae379_Supplemental_File

## Data Availability

The data underlying this article are available in the article and in its online supplementary material.
