# Supplementary material for: Albuminuria after induction treatment and kidney prognosis in ANCA-associated glomerulonephritis
Source: Clin Kidney J. 2024 Nov 23;18(1):sfae379. doi: 10.1093/ckj/sfae379 (PMC11852340; doi:10.1093/ckj/sfae379)
Supplement: sfae379_Supplemental_File [file sfae379_Supplemental_File.docx]

**Supplementary File**

**Albuminuria after Induction Treatment and Kidney Prognosis in ANCA-associated Glomerulonephritis**

Aglaia Chalkia^1^, Rachel Jones^1,2^, Rona Smith^1,2^, Lisa Willcocks ^2^, David Jayne^1,2^

^1^Department of Medicine, University of Cambridge, UK

^2^Vasculitis & lupus Clinic, Addenbrooke’s Hospital Cambridge, UK

1. **Supplementary Table 1:** Histopathological characteristics with comparison between groups with or without albuminuria at 6 months
2. **Supplementary Figure 1:** Prevalence of albuminuria beyond 6 months per Berden classification
3. **Supplementary Table 2:** Albuminuria levels at 6 months
4. **Supplementary Figure 2:** Estimated Glomerular Filtration Rate (eGFR) increase from baseline among late progressers cohort
5. **Supplementary Figure 3:** Kaplan Meier analysis of end-stage kidney disease in the late progressers cohort according to albumin-creatinine ratio (ACR) levels at 6 months
6. **Supplementary Table 3.** Univariable Cox proportional hazard analyses for kidney survival (end-stage kidney disease)
7. **Supplementary Table 4.** Sensitivity analysis univariable Cox proportional hazard analyses for combined end-point (end-stage kidney disease or death) in the late progressors cohort.
8. **Supplementary Table** **5.** Sensitivity analysis multivariable Cox proportional hazard analyses for combined end-point (end-stage kidney disease or death) in the late progressors cohort

**Supplementary Table 1**. Histopathological characteristics with comparison between groups with or without albuminuria at 6 months

| Characteristics | ‘’Late progressers’’ ^a^  N=190 | Albuminuria ^b^ | | p value |
| --- | --- | --- | --- | --- |
|  |  | Yes  N=64 | No  N=111 |  |
| **Berden Classification ^c^**  Focal class % (n)  Mixed class % (n)  Crescentic class % (n)  Sclerotic class % (n) | 47 (88)  29 (54)  17 (32)  7 (14) | 19 (12)  40 (25)  30 (19)  11 (7) | 64 (70)  24 (26)  7 (8)  5 (6) | **<0.001**  **0.029**  **<0.001**  0.495 |
| **Glomeruli**  Glomeruli %, median (IQR)  Normal Glomeruli (%), median (IQR)  Global Sclerosis (%), median (IQR)  Cellular Crescents (%), median (IQR)  Fibrinoid Necrosis (%), median (IQR)  Fibrous Crescents (%), median (IQR) | 20 (14-94)  46 (21-66)  8 (0-20)  11 (0-36)  12 (0-37)  0 (0-0) | 18 (12-31)  22 (10-43)  9 (0-23)  28 (6-50)  22 (0-55)  0 (0-0) | 20 (14-27)  57 (39-72)  8 (0-19)  8 (0-25)  10 (0-30)  0 (0-0) | 0.751  **<0.001**  0.416  **<0.001**  **0.036**  0.921 |
| **Interstitial/tubular area**  Fibrosis/ atrophy   - Absent % (n) - Mild % (n) - Moderate % (n) - Severe % (n)   Tubulitis % (n)  Acute tubular injury % (n) | 59 (99)  23 (38)  10 (17)  8 (13)  13 (24)  64 (116) | 58 (29)  29 (10)  12 (6)  10 (5)  13 (7)  61 (34) | 60 (59)  23 (23)  10 (10)  7 (7)  15 (16)  67 (70) | 0.888  0.814  0.491 |
| **Vessels**  Arteritis % (n)  Arteriosclerosis % (n) | 17 (31)  48 (85) | 11 (6)  46 (25) | 22 (23)  48 (50) | 0.129  0.868 |

n, number; IQR, interquartile range. The group of ‘’albuminuria’’ was defined as ACR more than 300 mg/g at 6 months.

^a^ The early progressers (28 patients) were excluded

^b^15 missing values for ACR after induction treatment

^c^ 2 missing values for Berden Classification

**Supplementary Figure 1.** Prevalence of albuminuria beyond 6 months per Berden classification


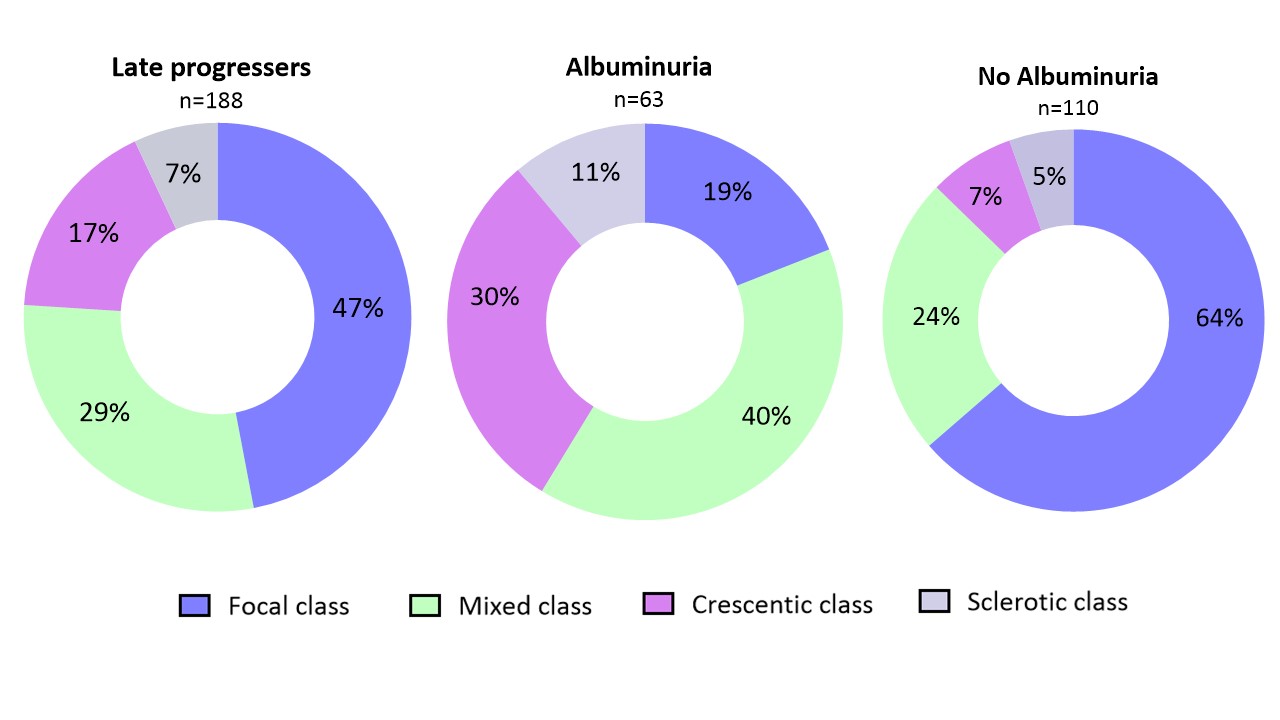


**Supplementary Table 2**: Albuminuria levels at 6 months

|  | ACR <30 mg/g | ACR 30-300 mg/g | ACR>300 mg/g |
| --- | --- | --- | --- |
| Patients, % (n) | 14 (20/144) | 43 (61/144) | 44 (63/144) |
| ESKD, n | 0 | 3 | 14 |

N; number, ESKD; end-stage kidney disease; ACR, urine albumin-to-creatinine ratio

**Supplementary Figure 2:** Estimated Glomerular Filtration Rate (eGFR) increase from baseline among late progressers cohort. (A) Estimated marginal means (EMMs) ± SEM (standard of error) eGFR increase by ACR levels over 5 years and (B) eGFR increase from baseline per year over 5 years obtained by mixed effects model for repeated measures analysis with fixed effects ACR levels, time, ACR levels and time interaction as factors, and baseline eGFR and age as covariates. Box and whisker plot; the line represents the median value, and the length of the box reflects the interquartile range (IQR). The T-bars represents maximum and minimum values. **p<0.001. ns; no significant, albumin-creatinine ratio (ACR) at 6month; 1; ACR <30 mg/g, 2; ACR 30-300 mg/g, 3; ACR>300mg/g


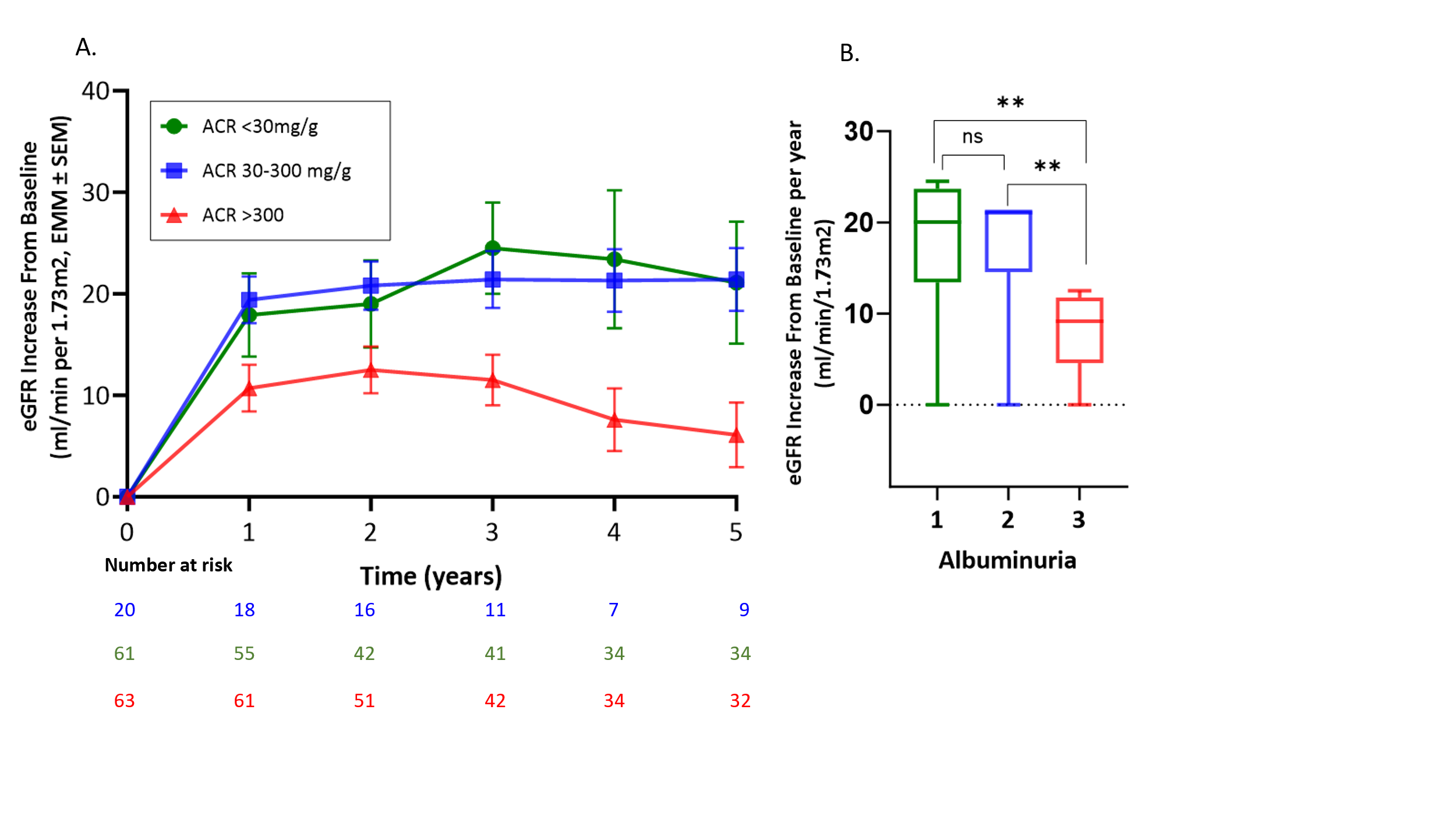


**Supplementary Figure 3:** Kaplan Meier analysis of end-stage kidney disease in the late progressers cohort according to albumin-creatinine ratio (ACR) levels at 6 months.


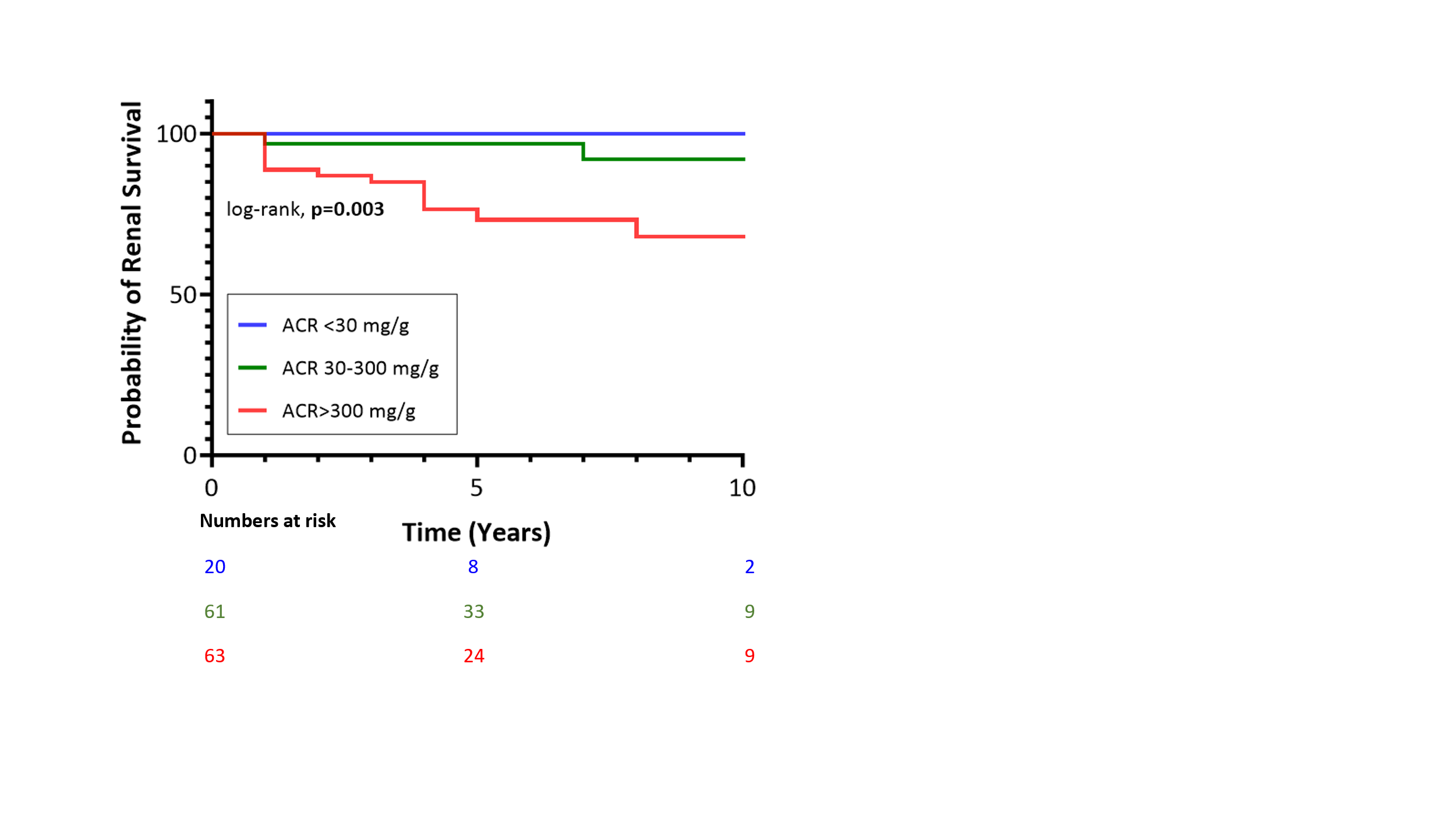


**Supplementary table 3.** Univariable Cox proportional hazard analyses for kidney survival (end-stage kidney disease) in the late progressors cohort.

| Variable | Univariable Cox regression | |
| --- | --- | --- |
|  | HR (95% CI) | p value |
| Albuminuria M6 | 8.87 (2.54-32.93) | **0.001** |
| ANCA  PR3 (Ref. MPO) | 1.24 (0.53-2.87) | 0.612 |
| Age | 1.02 (0.99-1.06) | 0.157 |
| Gender  Female (Ref. male) | 0.77 (0.32-1.84) | 0.564 |
| eGFR at diagnosis | 0.93 (0.89-0.97) | **0.001** |
| Haematuria M6 | 2.25 (0.83-6.11) | 0.110 |
| Berden classification  Focal (Ref.)  Mixed  Crescentic  Sclerotic | Reference  2.4 (0.82-6.99)  1.8 (0.51-6.52)  2.9 (0.59-14.78) | 0.107  0.350  0.184 |
| Induction treatment  RTX (Ref.)  CYC  RTX+CYC | Reference  0.34 (0.12-0.98)  1.3 (0.43-3.93) | **0.046**  0.627 |
| Normal glomeruli (%) | 0.97 (0.96-0.99) | **0.014** |
| Cellular crescents (%) | 1.0 (0.98-1.01) | 0.981 |
| Global sclerosis (%) | 1.02 (1.00-1.04) | **0.017** |
| Interstitial fibrosis  Absent (Ref.)  Mild  Moderate  Severe | Reference  1.30 (0.43-3.92)  1.42 (0.30-6.60)  3.17 (0.84-11.96) | 0.637  0.655  0.087 |

M6; month 6, HR; hazard ratio, CI; confidence internals, Ref.; reference, ANCA, Antineutrophil Cytoplasmic Antibody; MPO, Myeloperoxidase; PR3, proteinase 3; eGFR, estimated glomerular filtration rate; RTX, rituximab; CYC, cyclophosphamide.

The group of ‘’albuminuria’’ was defined as ACR more than 300 mg/g at 6 months.

**Supplementary Table 4.** Sensitivity analysis univariable Cox proportional hazard analyses for combined end-point (end-stage kidney disease or death) in the late progressors cohort.

| Variable | Univariable Cox regression | |
| --- | --- | --- |
|  | HR (95% CI) | p value |
| Albuminuria M6 | 2.02 (1.03-3.85) | **0.038** |
| ANCA  PR3 (Ref. MPO) | 0.94 (0.51-1.72) | 0.854 |
| Age | 1.05 (1.02-1.08) | **<0.001** |
| Gender  Female (Ref. male) | 0.77 (0.41-1.43) | 0.416 |
| eGFR at diagnosis | 0.97 (0.95-0.98) | **0.001** |
| Haematuria M6 | 1.28 (0.65-2.52) | 0.460 |
| Berden classification  Focal (Ref.)  Mixed  Crescentic  Sclerotic | Reference  1.95 (0.95-4.02)  0.91 (0.33-2.53)  2.69 (0.97-7.48) | 0.068  0.869  0.057 |
| Induction treatment  RTX (Ref.)  CYC  RTX+CYC | Reference  0.46 (0.22-0.99)  1.04 (0.40-2.71) | **0.048**  0.925 |
| Normal glomeruli (%) | 0.98 (0.97-1.00) | 0.058 |
| Cellular crescents (%) | 0.99 (0.97-1.00) | 0.187 |
| Global sclerosis (%) | 1.02 (1.01-1.04) | **<0.001** |
| Interstitial fibrosis  Absent (Ref.)  Mild  Moderate  Severe | Reference  1.40 (0.64-3.04)  1.34 (0.45-4.00)  4.36 (1.78-10.70) | 0.396  0.597  **0.001** |

M6; month 6, HR; hazard ratio, CI; confidence internals, Ref.; reference, ANCA, Antineutrophil Cytoplasmic Antibody; MPO, Myeloperoxidase; PR3, proteinase 3; eGFR, estimated glomerular filtration rate; RTX, rituximab; CYC, cyclophosphamide.

The group of ‘’albuminuria’’ was defined as ACR more than 300 mg/g at 6 months.

**Supplementary Table 5.** Sensitivity analysis multivariable Cox proportional hazard analyses for combined end-point (end-stage kidney disease or death) in the late progressors cohort.

| Variable | Adjusted Model 1  (32 events) | | Adjusted Model 2  (36 events) | | Adjusted Model 3  (30 events) | |
| --- | --- | --- | --- | --- | --- | --- |
|  | HR  (95% CI) | p value | HR  (95% CI) | p value | HR  (95% CI) | p value |
| Albuminuria | 1.57  (0.71-3.47) | 0.258 | 2.04  (1.02-4.05) | **0.041** | 1.41  (0.60-3.30) | 0.423 |
| eGFR at diagnosis | 0.97  (0.96-0.99) | **0.031** | 0.98  (0.96-1.00) | 0.078 | 0.98  (0.96-1.00) | **0.048** |
| Age |  |  | 1.04  (1.01-1.07) | **0.004** |  |  |
| Berden classification  Focal (Ref.)  Mixed  Crescentic  Sclerotic | Reference  1.19  (0.48-2.97)  0.48  (0.13-1.75)  1.55  (0.45-5.32) | 0.699  0.272  0.487 |  |  |  |  |
| Normal glomeruli (%) |  |  |  |  | 0.99  (0.97-1.01) | 0.666 |

Albuminuria defined as urine albumin-to-creatinine ratio>300 mg/g at 6months; HR, hazard ratio; CI, confidence internals, eGFR, estimated glomerular filtration rate; Ref, reference
